# Treatment of Cholera

**Published:** 1849-06

**Authors:** 


### TREATMENT OF CHOLERA.

Chloroform inhalations were used by Richet at the Hopitaux des Cliniques, but without any satisfactory results. Applications of the same were made without success by M. Vernois, at the Hopital St. Antoine, but he has used wtth advantage, in some cases, a combination of chloroform and laudanum.

Dr. Fenner treated the disease, in New Orleans, upon the following plan. "A large sinapism to the abdomen, smaller ones to the extremities, friction with spirits of turpentine to the legs. The following medicines internally:

℞. Calomel ʒss.

Sulph. morphia, gr. ss. M.

of this he gave about two-thirds immediately, and soon afterwards the following mixture:

℞. Spts. camphor, ℥jss.
Spts. lavender comp.
Tr. opii, *aa* ʒj. M.

Two teaspoonsful of this were given, and repeated after every stool."

Dr. Fenner restricted his patients to a very limited allowance of cold water. Where he suspected the presence of indigestible food in the stomach, he gave an emetic of salt and mustard. "As to the value of calomel in the treatment of cholera," he says, "I am decidedly of opinion that it is inestimable." He adds, "Dr. J. J. Kerr, of this city, has treated with complete success several cases in the *collapsed* condition, with large doses of calomel. He throws in from sixty to one hundred grains of this medicine every one or two hours, until vomiting and purging are checked, and dark feculent discharges are produced. He likewise advises a suppository of opium, as auxiliary to the treatment. From 500 to 1,000 grains of calomel have been administered in the collapsed stage, and the only bad effect has been a slight salivation."

Dr. Buchanan, of Nashville, used immense doses of opium and camphor in some of his last cases. Two of these were of a desperate character, but were recovered by the use of these agents. To one patient he administered forty grains of opium in twenty-four hours, and to another forty-four grains, in the same period.

